# Minimally Invasive Surgery for Pediatric Tumors – Current State of the Art

**DOI:** 10.3389/fped.2014.00048

**Published:** 2014-06-03

**Authors:** Jörg Fuchs, Luana Schafbuch, Martin Ebinger, Jürgen F. Schäfer, Guido Seitz, Steven W. Warmann

**Affiliations:** ^1^Department of Pediatric Surgery and Pediatric Urology, University Children’s Hospital Tübingen, Tübingen, Germany; ^2^Department of Pediatric Oncology, University Children’s Hospital Tübingen, Tübingen, Germany; ^3^Department of Clinical and Interventional Radiology, University Children’s Hospital Tübingen, Tübingen, Germany

**Keywords:** solid tumors, tumor resection, minimally invasive surgery, laparoscopy, thoracoscopy

## Abstract

During recent years, minimally invasive surgery (MIS) has become the standard approach for various operations in infants and children. This also holds true for surgery in children with solid tumors. Meanwhile, more and more oncological biopsies and resections are being performed laparoscopically or thoracoscopically. Despite its increasing role in pediatric tumor surgery, the different national and international multicenter trial groups have not yet implemented MIS within guidelines and recommendations in most of the current treatment protocols. An increasing number of reports describe a potential role of MIS in the different entities of pediatric surgical oncology. Over the time, there has been a diverse development of this approach with regard to the different neoplasms. The aim of this article is to give an overview and to describe the current state of the art of MIS in pediatric solid tumors.

## General Considerations

The outcome of children suffering from solid tumors has been dramatically improved over the past 30 years. Several reasons are responsible for this fact. One major factor in this regard is the treatment of patients according to guidelines and protocols established by multicenter trial groups. With great efforts, the different groups have realized a constant development and improvement of treatment modalities for every entity. Today, the most relevant groups with the largest experiences are the International Society of Pediatric Oncology (SIOP) in Europe and overseas, the Children’s Oncology Group (COG) in the US and North America, the Society of Pediatric Oncology and Hematology (GPOH) in Germany, Austria, and Switzerland, and the Japanese Pediatric Liver Tumor Study Group (JPLT) in Japan. A common perception of these groups is the continuing improvement of patient outcomes through combined therapy approaches integrating chemotherapy, surgery, and/or radiotherapy into concerted treatment concepts (1).

Besides these combined approaches, there has been a relevant advancement and improvement of every single portion of the different treatment aspects. For the surgical field, there are numerous examples for such advancements.

Minimally invasive surgery (MIS) has become the standard treatment approach for many operations within all age groups in pediatric surgery (2–5). From an early phase of this development on, oncological surgical procedures were performed minimal-invasively in children. Early reports described several limitations of the method because of various reasons (6–8). With an increase of frequencies, there was a growing knowledge on MIS in children with solid tumors. Nevertheless, several relevant issues and limitations have constantly been discussed, which make a general judgment of the method difficult.

The characteristics of the different frequent pediatric solid tumors are largely varying. This especially concerns tumor biology, affected organs, growth pattern, treatment concepts, and other factors. The heterogeneity of the different entities requires differing treatment concepts; accordingly, the role of surgery differs to a large degree from entity to entity. Tumor biopsy represents an example in this regard. In some tumors, a biopsy is required; sometimes biopsy is allowed but not mandatory, and sometimes it must not be performed. Some children with tumors receive neoadjuvant chemotherapy; some patients undergo upfront tumor resection. In some tumors, complete microscopic resection is of prognostic significance, in some tumors minimal residual disease can be accepted. Because of this differing role of surgery in the different entities, it is not possible to attribute a common objective to a single surgical approach such as MIS.

Reports of minimally invasive surgical procedures are increasingly observable. In a relevant number of cases, pediatric surgeons with a main focus of their work being MIS and not oncological surgery are performing these procedures. However, the emphasis of surgery in children with solid tumors lies not on the feasibility but on the strict adherence to oncological principles. It has therefore been repeatedly postulated that surgeons performing MIS for solid tumors in children should have experience in both fields, MIS and oncological surgery. This aspect is even more relevant since guidelines within the current treatment protocols of multi center trials are regularly lacking recommendations on MIS. These guidelines were often established some years ago when MIS did not play a prominent role in the surgical treatment of children with solid tumors (9, 10). Also, no randomized controlled trials or controlled clinical trials have been conducted so far analyzing surgical and oncological outcomes of MIS in pediatric tumor patients compared to open surgery (11). Several study protocols are currently in the process of being renewed. Therefore, it is a central task to surgeons involved in these proceedings to establish guidelines for MIS in children suffering from respective neoplasms to be implemented in the treatment protocols by the steering groups.

Despite all efforts of engaged surgeons to assess the role of MIS in pediatric surgical oncology and to have patients benefit from its advances, there are still relevant limitations of the approach. Some disadvantages of MIS are known from other surgical fields such as the tactile deficit and the limited visualization of lesions, which are not located on an organ surface. However, there are additional constraints that are especially relevant for oncological operations. It is a known fact that the radiological findings before surgery do not always correspond well with the intraoperative aspects. For example, in surgery for lung metastases, imaging possibly underestimates the number of pulmonary nodules to a relevant degree (12, 13). Also, it can be difficult in some cases to estimate the delineation of borders between tumor and unaffected tissue and thus to distinguish between infiltration and displacement of organs. A potential consequence during surgery is to leave tumor behind or to damage healthy organs with an increased risk for organ dysfunction or other co-morbidities. Under such circumstances, conversion to the open approach should not be judged as complication but rather as thoughtful course of action. Operating surgeons should always keep the safety of the patients and the correctness of the operations in mind.

In the following, the current state of the art regarding MIS in children with solid tumors will be displayed focusing on the entities within the thorax or abdomen, that are most commonly operated using MIS as approach.

## Thoracic Tumors

Primary thoracic tumors are usually surgically classified according to their anatomical occurrence. Most common tumors of the anterior mediastinum are lymphoma, thymus processes, angioma, lymphangioma, sarcoma, and germ cell tumors. Most common neoplasms of the middle mediastinum are lymphoma and angioma and most common tumors of the posterior mediastinum are neurogenic tumors and sarcoma (14). MIS is most relevant for biopsy in these tumors. Thoracoscopic resections are mostly carried out in lesions of the posterior mediastinum (14–17).

Technical challenges of MIS for thoracic tumors are relevant. The use of single lung ventilation is generally recommended, however, it can be difficult to establish in small children and infants. Handling and removal of the resected specimen from the thoracic cavity should be performed using a retrieval bag; in some cases, a mini-thoracotomy might become necessary for this purpose.

In small children, MIS for thoracic pathologies is possibly limited because of a small working space. Also, mechanical ventilation during surgery can be relevantly hampered because of lung compression and increased intra-thoracic pressure. Finally, the intraoperative carbon dioxide uptake might represent an anesthesiological issue that needs further evaluation.

### Thoracic neurogenic tumors

Minimally invasive surgery for resection of primary thoracic tumors is most often executed for neurogenic tumors. These tumors usually arise along the vertebral column within the posterior mediastinum and often show a benign or moderately aggressive biological behavior (14). The group of thoracic neurogenic tumors in children includes neuroblastoma, ganglioneuroma, Schwannoma, neurofibroma, and ganglioneuroma. They regularly appear quite specific on imaging. MIS has become a commonly used approach in these cases for biopsy and resection. Several studies could demonstrate equally good oncological and surgical outcomes in respective patients compared to open surgery (18–21). Surgical and clinical outcomes seem superior in comparison to the open approach because patients are spared thoracotomies or sternotomies. Long-term survival of children undergoing thoracoscopic resection of mediastinal neurogenic tumors seems higher when compared with those reported for other primary neurogenic tumor localizations (20, 21). However, extensive resection with relevant risk of functional damage should not be performed in benign cases, irrespective of the treatment approach (14).

### Thoracic teratoma

Approximately 4% of all germ cell tumors are located within the thorax, especially within the anterior mediastinum (22). Benign differentiated teratomas are usually well-confined and consist of components of all three germinal layers.

The well-known tumor markers AFP and beta-HCG are indicators for malignant components within the tumors (yolk-sac or chorio-carcinoma). Complete surgical resection represents the most important prognostic factor for patients with malignant germ cell tumors of the thorax. Moreover, malignant thoracic germ cell tumors often present with a local stage T2 because of infiltration of the surrounding tissue (22). For these reasons, the use of MIS for resection has to be judged critically within this group.

### Pulmonary metastases

Thoracoscopy has been suggested for approaching various conditions of lung metastases from solid tumors in children (16, 23–25). General recommendations proposed MIS as preferable tool for biopsy of lung nodules if tissue for biological information is needed without intention to completely remove all lesions visible on diagnostic imaging (23). Regularly, there is a relevant discrepancy between the number of radiologically detectable lung nodules and the respective intraoperative findings. This discrepancy becomes more and more relevant with the increase of lung nodule numbers (12, 13). Important limitations have to be taken into consideration with regard to MIS for lung metastases. This especially concerns the lack of tactile abilities and the inability to intraoperatively visualize lesions that are deeper within the parenchyma. Different tools have been suggested in order to improve the intraoperative detection during MIS of lung metastases that are not visible on the lung surface. These approaches include preoperative labeling (coils, coil wires, color dye, or radionuclides) as well as minimally invasive thoracoscopic ultrasound (26–29). Together with constantly improved diagnostic procedures, these tools make pulmonary metastases becoming more and more susceptible to MIS. However, in some conditions the completeness of pulmonary metastasis resection is associated with a high prognostic impact, as it has been demonstrated for nephroblastoma (30). As consequence, the use of MIS for resection of pulmonary metastases should only be performed according to respective treatment guidelines within the trial protocols and considering the biological background of the tumors.

## Abdominal Tumors

Laparoscopy is regularly performed for various conditions of abdominal solid malignancies in children. Early reports have attempted to define the role of MIS in this group. Up to date, there is no definite consensus although most authors distinguish between a relevant role of MIS for biopsies as well as a limited role for tumor resections (8, 31). Main advantages of MIS seem to be a reduced time to postoperative mobilization and feeding as well as reduced time to a subsequent chemotherapy. Some authors reported on complex minimally invasive procedures for resection of malignancies in adults and in children. Examples such as laparoscopic anatomical liver resections or complex procedures on the pancreas and biliary tract in adults are indicating that there still is an ongoing development in that specific field (32–34).

The impact of MIS on tumor growth or biology has been an element of discussion for a while delivering inconsistent observations in preclinical models. This especially concerns the use of carbon dioxide (35, 36). However, after being used for a relevant time with respective follow-up, clinical experiences with laparoscopy for pediatric abdominal malignancies seem to indicate that there are no relevant differences in outcome compared to open surgical procedures. However, principles of oncological surgery have to be strictly respected using MIS. Especially because of a broad variety of characteristics from entity to entity there is still an ongoing need for a definite evaluation of MIS in affected children.

The modality of tumor removal from the abdominal cavity is varying relevantly between different surgeons and obviously depends on several factors such as tumor size and volume in relation to the patient, tumor histology, and patients’ conditions. The use of a retrieval bag often in combination with a small laparotomy (mostly Pfannenstiel incision) is regularly carried out. Port-site metastases seem to be rather rare events if enough caution is given to the handling of the tumors (37, 38).

### Adrenal tumors

Abdominal adrenal tumors display a wide variance of histological, biological, and anatomical features. Most common pathologies include neuroblastoma, ganglioneuroma, adenoma, and pheochromocytoma (39).

Adrenal neurogenic tumors are regularly well-confined and not infiltrating or encasing substantial organs and structures. In such cases, MIS for biopsy and/or resection has been established as safe alternative to open surgery, especially if the tumor size is not excelling a certain limit (40–42). However, a complex spatial relationship to vital structures is possible and represents a relevant obstacle for MIS.

Neuroblastomas in small infants do often not seem to need surgical treatment. In such cases, even if they appear resectable without great efforts, surgery contains an additional risk for morbidity. The proposed primary approach in this subgroup of patients is expectant observation. Secondary surgery becomes necessary in this setting in case of a 50% increase in the volume of the mass, urine catecholamine values, or an increase in the homovanillic acid to vanillylmandelic acid ratio >2. Even MIS seems not to be justified as primary approach in these selected cases because event-free survival and overall survival is excellent when patients undergo the cited approach of expectant observation as primary therapy (43).

Adrenocortical carcinoma has an exceptional position within the group of adrenal tumors. They are associated with relevantly inferior oncological outcomes after MIS because of a higher rate of relapses after resection. Although feasible and tempting in many cases, laparoscopic biopsy or resection should not be attempted in patients with tumors suspicious for or known to be adrenocortical carcinoma (44, 45). Often, it is possible to distinguish adrenocortical carcinomas from other adrenal tumors because of their radiological and biological behavior (increased hormonal activity and associated clinical signs of hormonal excess). A thorough pre- and perioperative workup is therefore essential in these patients (45).

The use of MIS in para-vertebrally located neuroblastoma has not been reported in large series. These tumors are usually complex in their anatomical constellation, which is commonly reflected by their risk stratification using image-defined risk factors (46). Therefore, they are the primary domain of open surgical approaches.

### Renal tumors

Complete surgical resection is one of the strongest predictors for outcome in Wilms tumor surgery. Together with the complete removal of all neoplastic compounds, a sufficient lymph node sampling is a main goal of surgical treatment in affected children (47, 48). Laparoscopy is increasingly used for tumor nephrectomy in nephroblastoma patients (49, 50), however, a systematical analysis from a multicenter trial has been lacking for a long time. The SIOP Renal Tumor Study Group (RTSG) has recently presented the first according assessment, which demonstrated a comparable surgical and oncological outcome as in open surgery (51). However, operating surgeons had above-average experiences with Wilms tumor surgery in that study. The insufficient discipline of lymph node sampling was one major aspect of concern to the investigators. Another important issue is the tumor localization within the organ. Often small and polar tumors are described as being resected laparoscopically. However, such lesions are possibly susceptible for nephron-sparing surgery (NSS). The SIOP RTSG Surgical Panel therefore formulated the recommendation that MIS and NSS should not be regarded as competing approaches: if a tumor can be resected using NSS then this is the preferred method of choice. The according guidelines are formulated within the treatment protocol. If a decision for tumor nephrectomy has been established, then the surgeon might choose MIS as approach (52). The principles of Wilms tumor surgery remain valid just as in open surgery.

A technical problem possibly arises because of the tumor size. Large specimens might be difficult to handle intra-abdominally with a substantial risk of tumor rupture. However, this has to be avoided since it is associated with a local up-staging and subsequent need for irradiation. The ipsilateral vertebral border seems to represent a limitation in that regard.

### Ovarian tumors

Complete resection is mandatory for malignant pediatric ovarian tumors. Diagnosis of ovarian tumors in girls is based on clinical features (age and hormonal status), imaging, and tumor marker levels. In the case of benign tumors, preservation of healthy ovarian tissue is crucial. When the lesion is malignant, laparoscopy has a defined role for establishing an exact diagnosis and for staging. However, MIS should only be cautiously applied for resection of malignant pediatric ovarian tumors since laparotomy via a supra-pubic approach has relevant advantages in ensuring a safe treatment of the lesion by avoiding any risk of tumor spillage (53, 54).

### Pancreatic tumors

Tumors of the pancreas are rare in children. In case of difficulties to establish a diagnosis, MIS might be considered for tumor biopsy. However, a possible negative impact of MIS through tumor spillage during biopsy of highly malignant lesions (blastoma, carcinoma) has to be strongly taken into consideration. Accordingly, reports on laparoscopy for pancreatic masses almost exclusively exist for pseudopapillary tumors (55–57). Despite its challenging but well-executable technical aspects, there exist relevant concerns with MIS for pseudopapillary tumors of the pancreas in children because of tumor recurrence after intraoperative cell spillage (58).

## Conclusion

Minimally invasive surgery is increasingly being used as surgical approach in children with solid tumors. Indications and decision-making processes are still largely varying. Furthermore, there is a lack of surgical guidelines regarding MIS for the different tumors within current treatment trials. Therefore, systematical assessments are necessary to define standardized proceedings. Retrospective studies should be used at this stage in order to obtain background information through which investigators will be enabled to initiate prospective analyses in the future. The protocols of the various multicenter trial groups around the world are probably the most promising platforms for integrating such prospective studies in a comprehensive data acquisition and interpretation. The surgical panels of the various study groups should make it their goal to formulate guidelines for MIS in pediatric solid tumors within these protocols. Until such information is available, MIS for tumors in children should be used with a careful patient selection after thorough decision-making processes. An exceptional expertise in MIS and surgical oncology is essential for operating surgeons and treating centers in order to obtain acceptable treatment outcomes. MIS in pediatric tumor patients has to be executed within the established treatment regimens formulated by multicenter trial groups. The regular and defined integration of MIS within future treatment protocols is only justified if oncological and surgical results are at least on the same level as those obtained through open surgical procedures.

## Conflict of Interest Statement

The authors declare that the research was conducted in the absence of any commercial or financial relationships that could be construed as a potential conflict of interest.
